# Factors associated with accuracy of rapid HIV test performance in the Eastern Cape, South Africa

**DOI:** 10.4102/sajhivmed.v26i1.1641

**Published:** 2025-02-28

**Authors:** Amanda A. Mohlala, Edith Phalane, Claris Siyamayambo, Musa Jaiteh, Refilwe N. Phaswana-Mafuya

**Affiliations:** 1 South African Medical Research Council/University of Johannesburg Pan African Centre for Epidemics Research (SAMRC/UJ PACER) Extramural Unit, University of Johannesburg, Johannesburg, South Africa; 2 Department of Environmental Health, Faculty of Health Sciences, University of Johannesburg, Johannesburg, South Africa; 3 Strategic Evaluation Advisory Development (SEAD) Consulting, Cape Town, South Africa

**Keywords:** quality assurance, HIV, accuracy, proficiency testing, Eastern Cape

## Abstract

**Background:**

Testing for HIV using rapid test devices assists in determining HIV status and ascertains if treatment is required. Rapid HIV quality assurance (QA) training is conducted to ensure accuracy of test results in non-laboratory settings.

**Objectives:**

This study aimed to determine the factors associated with the accuracy of rapid HIV testing among primary healthcare (PHC) workers who received HIV testing QA training in the Eastern Cape, South Africa.

**Method:**

A cross-sectional analytical study design was used to purposively recruit 167 PHC workers with prior training on HIV testing QA. Data were collected in 2022 using a self-administered questionnaire. STATA version 17.0 was used for data analysis.

**Results:**

Tester accuracy measured by a proficiency testing (PT) score of greater than 80% was achieved among 64.7% of the testers. Comprehensive HIV QA training was significantly associated with a PT score of greater than 80% (*P* = 0.001). On multivariable analyses, a PT score of greater than 80% was less likely with rural facilities (adjusted odd ratios [aOR] = 0.56, 95% confidence interval [CI]: 0.36–0.92, *P* = 0.020), Grade 12 education (aOR = 0.40, 95% CI: 0.19–0.85, *P* = 0.017), mentorship (aOR = 0.55, 95% CI: 0.35–0.85, *P* = 0.008), and attitudes towards inaccurate HIV results (aOR = 0.13, 95% CI: 0.02–0.82, *P* = 0.03); while implementation of QA processes (aOR = 3.94, 95% CI: 1.22–12.74, *P* = 0.022) and elements of QA in the HIV Testing Services register (aOR = 4.93, 95% CI: 1.45–16.74, *P* = 0.011) were associated with a PT score of greater than 80%.

**Conclusion:**

Exposure to comprehensive rapid HIV QA training were associated with higher tester accuracy. A framework for QA training is required for standardisation of training in the country.

**What this study adds:** The study evaluated factors associated with the accuracy of rapid HIV testing by primary healthcare workers. Higher accuracy was associated with quality assurance processes.

## Introduction

By the end of the year 2021, South Africa reported 210 000 new HIV infections and 8.2 million people living with HIV (PLHIV).^1,2^ Rapid HIV testing plays a key role in the continuum of care for newly HIV-diagnosed individuals to be initiated on treatment, with the aim of eventual viral suppression. Rapid Test Devices (RTDs) are relatively robust, and performance of these tests is similar to HIV laboratory-based assays with generally acceptable sensitivity and specificity. Despite this, Kosack et al. reported that performance at testing sites and by non-technical staff is challenging and reports in the literature generally demonstrate a lower sensitivity and specificity.^3^ The South African HIV Testing Services (HTS) policy guidelines put emphasis on expansion of HTS to the high-risk key populations.^4^ The provision of rapid HIV testing by non-professional staff using RTDs was approved and gazetted by the National Department of Health (NDOH) in 2010.^5^ The Gazette allowed non-professionals to provide HIV rapid testing following adequate rapid HIV tester quality assurance (QA) training. The 2017, annual Stepwise Process for Improving the Quality of HIV Rapid Testing (SPI-RT) assessments revealed that the Eastern Cape performed below the national average for HIV rapid testing QA indicators. The province scored 61.5% compared to the South African average of 78.4%. Additionally, the staff training and competency indicator was significantly lower, with a score of 40% compared to the national average of 61.2%.^6^

Even though HIV RTDs are easy to perform where testers are inadequately trained and lack experience in testing, the devices may present with limitations to achieving accurate and reliable test results. For example, test kit instruction leaflets often show examples of non-complex results. Therefore, testers without comprehensive training and experience might not have the required skills to interpret more complex results, such as discrepant or invalid results. Studies have shown these challenges may occur more often among counsellors who have not received adequate training.^6,7^ Recommendations from the World Health Organization (WHO) state that training should be augmented by other factors, such as mentorship and supervision of testers, policy development, and adherence to testing procedure to ensure quality of testing and successful scale-up of an HTS programme.^8^

A study conducted in a rural district in KwaZulu-Natal (KZN) showed improved sensitivity of 98% and specificity of 99.6% following comprehensive training and practical clinic-based experience of lay counsellors, which enhanced performance and adherence to testing procedures.^9^ The same study compared quality of testing between lay counsellors and laboratory-based testing. The lay counsellors attended a 10-day comprehensive HTS training followed by job shadowing of facility-based counsellors and training by laboratory personnel prior to field work. Study data showed that the lay counsellors’ and laboratory results showed a concordant rate of 99.5%.^10^

The introduction of the Universal Test and Treat (UTT) approach for care and treatment has put a stronger focus on the quality of HIV testing.^11^ Ideally, RTDs can produce accurate and reliable results. However, as stated above, limitations to producing such results do exist and there have been reports on poor quality of HIV testing, including operator-related errors, inappropriate test kit storage, inadequate training, and lack of supervision.^9,10^ Proficiency testing (PT) is a method where blinded samples are issued by the National Reference Laboratory (NRL). In South Africa, the National Health Laboratory Services (NHLS) serves as the NRL and offers a PT Scheme (referred to as surveys) to almost 4000 facilities twice a year.^12^ The WHO defines external quality assurance (EQA) as a system that is designed to objectively monitor the performance of a testing site, including testers and all systems used for testing, through an external agency.^8^ For facilities that routinely perform HIV testing, EQA programmes such as PT for rapid HIV testing are a priority to ensure the accuracy and reliability of test results.^13^ The NHLS has set the minimum acceptable average score of 80%.^12^ This score can be used to establish the level of accuracy of test results and can assist in identifying the training requirements of testers who did not perform satisfactorily.^8^

The study aimed to determine the factors associated with the accuracy of rapid HIV testing among primary healthcare (PHC) workers who received HIV testing QA training in a selected district in the Eastern Cape, South Africa.

## Research methods and design

### Study design

A cross-sectional analytical study design was conducted to determine the factors associated with the accuracy of rapid HIV testing among PHC workers who received HIV testing QA training in the Eastern Cape province of South Africa.

### Study setting

This study was conducted in rural, semi-urban and urban facilities in one of the selected health districts of the Eastern Cape. The district has four health sub-districts with 161 PHC facilities. A total of 35 facilities were purposively selected for the study based on the inclusion criteria. The selection criteria of facilities were based on the HIV testing high-burden facilities (> 1000 total patients remaining on antiretroviral therapy [ART]), and the selection was made in consultation with the district management team.

### Study population and sampling strategy

Inclusion criteria were healthcare workers (HCW) who were 18 years and older, linked to a PHC facility, conducting HIV rapid tests at facilities, and who were exposed to any form of rapid HIV test training. The study population for this research consisted of lay counsellors and nurse clinicians at 35 PHC facilities in the selected district who are involved in conducting rapid HIV tests as part of the HTS. Facilities with > 1000 total patients remaining on ART were included in this study. The district has a total population of 200 testers based in the selected healthcare facilities. Clinicians and lay counsellors are mandated to conduct rapid HIV tests at facility level.

The sample size was calculated using Epi Info version 7.2. (2016; US Centres for Disease Control and Prevention, Atlanta, Georgia, United States). A two-sided confidence interval (CI) of 95%, an acceptable error margin of 5% and a power odd ratio of 80% was used to calculate the sample size for the study. A 25% contingency was added for multivariate data analysis and the sample size was 167 (134 + 33).

A multistage sampling technique was used, with one health district chosen at random first. This was followed by a purposive selection of 35 facilities out of the 161 PHC facilities in the selected health district. Then the 167 study participants were also purposively selected from the 200 testers. This sampling strategy was utilised to ensure easy accessibility, geographical proximity, willingness to participate in the study and availability.

### Data collection

A self-administered questionnaire (SAQ) was administered to determine the relationship between rapid HIV tester QA training and accuracy of rapid HIV test performance among HCW, and to describe the factors and tester characteristics that affect accuracy of test performance. Data were collected during July 2022 and October 2022. The participants included in the study were exposed to the rapid HIV QA training curricula presented in English. The prerequisite for participation was proficiency in English. Therefore, the questionnaire was administered in English and not translated into the local language. The PT results achieved by the participants in the previous survey were used to determine the level of accuracy of test performance. For testers that routinely perform HIV testing, PT offers an objective evidence of testing quality. The minimum acceptable average score for accuracy of test performance is 80%. The SAQ was adapted from the Human Science Research Council (HSRC) ‘Assessment of HIV Counselling and Testing Services in South Africa: HIV Tester Questionnaire’.^14^ and utilised to collect data from HCW who are trained and conduct rapid HIV testing at facility level (Online Appendix 1). To ensure the validity of the study questionnaire, a pilot study population was selected to represent the broader study population (5% of the study population, *n* = 8) by choosing pilot facilities in close proximity to the study area, thereby reflecting comparable conditions and characteristics. The pilot study also allowed for evaluation of the reliability of the data collection tool. The pilot was conducted at an urban facility, to assess the instrument’s capacity to accurately capture data across all sections of the questionnaire. Additionally, the questionnaire was tested at a rural facility to account for variability in environmental and contextual factors.

The study variables were conceptualised to develop the questionnaire. The dependent variable was PT scores that are above 80% as a measure of accuracy of rapid HIV test performance or below 80% as a measure of inaccurate test result. This variable was used as a continuous variable and was coded 1 and 2. The questionnaire was also structured to collect information on demographics, knowledge, practices, and attitude factors that may contribute to accuracy of rapid HIV test performance. These responses were coded from 1 to 4.

### Data analysis

Data entry was done in Excel and checked for completeness and accuracy prior to exporting it to STATA version 17.0 (2021; StataCorp LLC, College Station, Texas, United States). The cleaning process entailed verification of each variable for unsuitable values. All data analysis was performed using STATA version 17.0 for overall and trend analysis. A chi-square test was used to test the association of the prevalence of accuracy of rapid HIV test performance and HCW that received HIV QA training. Descriptive analysis included frequencies and summary statistics. Frequencies were done for all categorical variables while summary statistics were done for continuous variables. Performance of logistic regression analysis was done to assess whether age, gender, occupation and facility type, and knowledge of rapid HIV testing processes would influence the accuracy of rapid HIV test performance among HCW. Bivariate logistic regression analysis was used to determine factors (socio-demographic factors, knowledge, attitudes, and practices) associated with accuracy of HIV testing. The bivariate logistic regression analysis was performed, and associations were determined using crude odds ratios (cOR), adjusted odd ratios (aOR), and *P*-value ≤ 0.05 to determine whether the association is statistically significant. Logistic regression analysis was done to measure if knowledge of rapid HIV testing processes influences attitude and practices of rapid HIV testing processes. Variables with a *P*-value of ≤ 0.05 and 95% CI from the bivariate logistic regression models were included in the final multivariate logistic regression model to determine significant factors associated with accuracy of HIV testing.

### Ethical considerations

Ethical considerations were observed during the research study. Ethics approval was obtained from the University of Johannesburg Faculty of Health Sciences Research Ethics Committee (study approval number: REC1550-2022).

#### Informed consent

All participants in the study were informed about the objectives of the research and what it seeks to achieve. Written informed consent was sought from the participants by requiring them to sign a consent form as their acceptance to participate voluntarily in the study.

#### Confidentiality

Unique numbers were used instead of participants’ identifying information such as names and ID numbers, thus their names will not appear in the final research. In addition, care was exercised in data handling to avoid losing data and leaks, which may expose the study participants.

#### Beneficence and non-maleficence

Participants of the study were treated with dignity and respect and the benefits of the study were explained. This included improvement of rapid HIV test performance and accuracy of results resulting in enrolment of the correctly diagnosed patients into care.

## Results

### Socio-demographic characteristics of the testers

A total of 167 participants were purposively selected to take part in the study. The participants were predominately women (*n* = 114; 68.3%). Each age group was represented; 51% of the participants were aged between 35 and 50 years, followed distantly by those aged 18–35 years (35.3%), and those above 50 years (13.8%). The study had a good mixture of participants having various educational qualifications: most participants had Grade 12 (Matric or Standard 10) education (37.1%), followed by post-Matric certificate or diploma (29.9%), Grade 11 or lower (Standard 9 or lower) (19.2%), and Baccalaureate Degree(s) (13.8%). Most participants were based in rural areas (44.3%), and the rest were in urban (43.7%) and semi-rural (12.0%) facilities. Study information was mainly gathered from clinics (73.1%), with hospitals comprising 19.2%, and mobile units 7.8%. The findings show that most participants were counsellors (56.9%), and nurse clinicians (42.5%) were also part of the study (see Table 1).

**TABLE 1:** Socio-demographic characteristics of testers (*N* = 167).

Variable	*n*	%
**Sex**		
Male	53	31.7
Female	114	68.3
**Age (years)**		
18–35	59	35.3
> 35–50	85	50.9
> 50	23	13.8
**Highest level of education**		
Grade 11 or lower (Standard 9 or lower)	32	19.2
Grade 12 (Matric or Standard 10)	62	37.1
Post-Matric certificate or diploma	50	29.9
Baccalaureate degree(s)	23	13.8
**Geographical location**		
Urban	73	43.7
Semi-rural	20	12.0
Rural	74	44.3
**Type of testing site**		
Clinic	122	73.1
Hospital	32	19.2
Mobile unit	13	7.78
**Occupation**		
Counsellor	95	56.9
Nurse clinician	72	43.1

### Prevalence of accuracy of test performance (> 80%)

**FIGURE 1:**
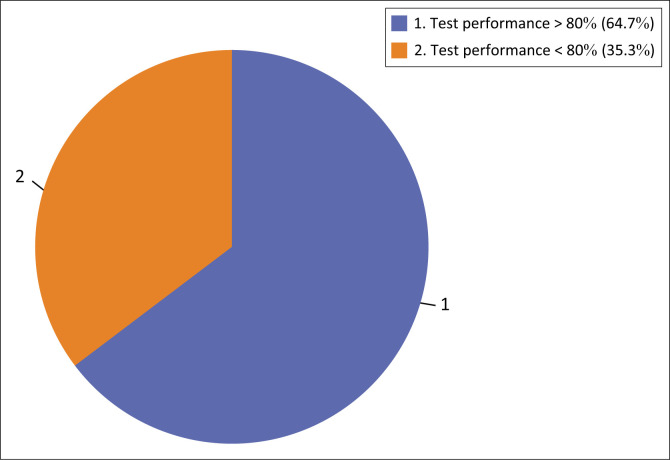
Prevalence of accuracy of test performance achieved amongst testers.

Tester accuracy levels measured by a PT score of greater than 80% were achieved among 64.7% of the testers (Figure 1).

### Association between prevalence of accuracy of rapid HIV test performance and healthcare workers that received rapid HIV quality assurance training

Of the 167 testers who participated and received feedback in the proficiency test survey, the results showed that 97.2% of testers who achieved high prevalence of accuracy of test performance (> 80%) were exposed to comprehensive rapid HIV QA training. Only 2.8% of participants who reported that the rapid HIV test training received did not address the QA principle of testing were able to achieve high prevalence of accuracy of test performance. Based on the chi-square test findings (Table 2), there was an association between attending training on rapid HIV QA practices and prevalence of accuracy of rapid HIV test performance using PT results among PHC workers (*P* = 0.001). However, based on the chi-square test findings, the results of the study also showed that there is no association between duration of employment and accuracy of rapid HIV test performance using PT results among PHC workers (*P* = 0.550).

**TABLE 2:** Association between prevalence of accuracy of rapid HIV test performance and healthcare workers that received rapid HIV quality assurance training (*N* = 167).

Variable	*n*	Test performance				*P*
		> 80%		< 80%		
		*n*	%	*n*	%	
**Have you ever attended training on rapid HIV Quality Assurance (QA) practices?**	-	-	-	-	-	0.001*
Yes	154	105	97.20	49	83.10	-
No	13	3	2.80	10	16.90	-
**How long have you been employed?**	-	-	-	-	-	0.550
0–6 months	5	2	1.85	3	5.08	-
> 6–12 months	23	14	12.96	9	15.25	-
> 12–18 months	19	14	12.96	5	8.47	-
> 18 months	120	78	72.22	42	71.19	-

* , The Chi-square statistic is significant at the 0.05 level.

### Association of socio-demographic factors associated with accuracy of rapid HIV test performance

The multivariate analysis in Table 3 did not demonstrate that gender and age of a participant were significantly associated with accuracy of rapid HIV test performance (gender: 95% CI: 0.49–1.97, *P* = 0.958; and age: 95% CI: 0.52–1.99, *P* = 0.966). However, a negative association was observed between lower accuracy of test performance and participants who had Grade 12 (Matric) (95% CI: 0.19–0.85, *P* = 0.017), and rural geographical location of participants (95% CI: 0.36–0.92, *P* = 0.020).

**TABLE 3:** Association of socio-demographic factors associated with accuracy of rapid HIV test performance.

Variable	Univariate analysis			Multivariate analysis		
	cOR	95% CI	*P*	aOR	95% CI	*P*
**Sex**						
Male†	-	-	-	-	-	-
Female	0.52	0.35–0.77	0.001*	0.98	0.49–1.97	0.958
**Age (years)**						
18–35†	-	-	-	-	-	-
> 35–50	0.55	0.35–0.85	0.008*	1.01	0.52–1.99	0.966
> 50	0.53	0.23–1.26	0.151	0.84	0.29–2.44	0.753
**Highest level of education**						
Grade 11 or lower (Standard 9 or lower)†	-	-	-	-	-	-
Grade 12 (Matric or Standard 10)	0.41	0.24–0.71	0.001*	0.40	0.19–0.85	0.017*
Post-Matric Certificate or Diploma	0.52	0.29–0.93	0.026*	0.48	0.10–2.18	0.339
Baccalaureate Degree(s)	0.44	0.18–1.06	0.068	0.40	0.07–2.43	0.322
**Geographical location**						
Urban†	-	-	-	-	-	-
Semi-rural	0.43	0.16–1.12	0.082	0.82	0.28–2.46	0.730
Rural	0.57	0.36–0.92	0.022*	0.56	0.36–0.92	0.020*
**Type of testing site**						
Clinic†	-	-	-	-	-	-
Hospital	0.52	0.25–1.09	0.082	1.17	0.48–2.85	0.731
Mobile unit	0.44	0.14–1.44	0.177	0.72	0.20–2.58	0.616
**Occupation**						
Counsellor†	-	-	-	-	-	-
Nurse Clinician	0.51	0.31–0.83	0.007*	1.14	0.25–5.16	0.861
Other	1.00	-	-	1.00	-	-

aOR, adjusted odds ratio; cOR, crude odds ratio; CI, confidence interval.

* , Significant at the 0.05 level.

† , Reference group; no values are generated when logic regression model is performed.

Multivariate data analysis did not reveal statistical significance when comparing association of accuracy of test performance to occupation. However, univariate data revealed statistical association between accuracy of test performance and nurses (95% CI: 0.31–0.83, *P* = 0.007). The type of testing site did not show any significant association with accuracy of test performance.

### Association of knowledge characteristics and accuracy of rapid HIV test performance

The multivariate analysis performed in Table 4 shows that a negative association was observed between accuracy of test performance and exposure to mentorship (95% CI: 0.35–0.85, *P* = 0.008). However, no statistical significance was observed between accuracy of test performance and knowledge of guidelines and policies of HTS (95% CI: 0.10–35.72, *P* = 0.669). Additionally, an association between accuracy of test performance and knowledge of monitoring the performance of test kits using independent quality control (IQC) was not statistically significant (95% CI: 0.02–6.01, *P* = 0.443).

**TABLE 4:** Association of knowledge characteristics and accuracy of rapid HIV test performance.

Characteristic	Univariate analysis			Multivariate analysis		
	cOR	95% CI	*P*	aOR	95% CI	*P*
**How often do you receive mentorship or technical support for rapid HIV processes**						
Monthly†	-	-	-	-	-	-
Quarterly	0.62	0.33–1.15	0.127	1.05	0.44–2.51	0.905
Bi-annually	0.80	0.21–2.98	0.739	1.43	0.33–6.25	0.637
Annually	0.62	0.26–1.48	0.280	1.23	0.41–3.65	0.708
I am not sure	0.48	0.23–0.98	0.044*	0.55	0.35–0.85	0.008*
**How well informed do you think you are concerning the guidelines and policies of HIV Testing Services?**						
Not at all informed†	-	-	-	-	-	-
Little informed	1.00	0.25–4.00	1.000	1.00	-	-
Fairly informed	0.70	0.39–1.27	0.241	2.91	0.14–61.09	0.492
Well informed	0.58	0.37–0.90	0.016*	1.00	-	-
Very well informed	0.27	0.11–0.67	0.005*	1.90	0.10–35.72	0.669
**Testing of independent quality control (IQC) samples to monitor performance of rapid HIV test kits is one of the key components of ensuring accurate and reliable results**						
Strongly disagree†	-	-	-	-	-	-
Disagree	1.00	0.006–15.99	1.000	0.50	0.01–28.31	0.734
Undecided or do not know	1.00	-	-	1.00	-	-
Agree	0.55	0.33–0.91	0.020*	0.32	0.02–6.01	0.443
Strongly agree	0.57	0.37–0.86	0.008*	0.42	0.02–7.81	0.562

aOR, adjusted odds ratio; cOR, crude odds ratio; CI, confidence interval.

* , Significant at the 0.05 level.

† , Reference group; no values are generated when logic regression model is performed.

### Association of attitude characteristics and accuracy of rapid HIV test performance

Table 5 illustrates the multivariate analysis of associations between attitude characteristics and rapid HIV test accuracy. The accuracy of test performance was negatively associated with participants who strongly agreed that inaccurate rapid HIV results is an issue of great importance in public health (95% CI: 0.02–0.82, *P* = 0.03). However, an association between accuracy of test performance and attitudes towards clear operational procedure on rapid HIV testing processes was not revealed (95% CI: 0.05–3.22, *P* = 0.386). A positive association was found between accuracy of test performance and participants who agreed that implementation of QA processes in rapid HIV testing is an extra burden of work (95% CI: 1.22–12.74, *P* = 0.022).

**TABLE 5:** Association of attitude characteristics and accuracy of rapid HIV test performance.

Characteristic	Univariate analysis			Multivariate analysis		
	cOR	95% CI	*P*	aOR	95% CI	*P*
**Inaccurate rapid HIV results is an issue of great importance in public health**						
Strongly disagree†	-	-	-	-	-	-
Disagree	4.00	0.45–35.79	0.215	0.21	0.02–2.51	0.219
Agree	0.53	0.35–0.80	0.002*	0.22	0.04–1.37	0.105
Strongly agree	0.36	0.19–0.68	0.002*	0.13	0.02–0.82	0.030*
**How important would it be for you to have a clear operational procedure on rapid HIV testing processes?**						
Not at all important†	-	-	-	-	-	-
Undecided or do not know	2.00	0.18–22.06	0.571	0.77	0.02–25.56	0.882
Important	0.56	0.32–0.96	0.035*	0.39	0.05–3.22	0.386
Very important	0.49	0.33–0.74	0.001*	0.40	0.05–3.25	0.393
**Do you think that implementation of QA processes in rapid HIV testing is an extra burden of work?**						
Strongly disagree†	-	-		-	-	-
Disagree	0.33	0.20–0.54	0.001*	0.58	0.22–1.53	0.270
Undecided or do not know	1.00	-	-	1.00	-	-
Agree	2.33	1.06–5.09	0.033*	3.94	1.22–12.74	0.022*
Strongly agree	0.70	0.27–1.84	0.469	1.56	0.44–5.49	0.488

aOR, adjusted odds ratio; cOR, crude odds ratio; CI, confidence interval; QA, quality assurance.

* , Significant at the 0.05 level.

† , Reference group; no values are generated when logic regression model is performed.

### Association of practice characteristics and accuracy of rapid HIV test performance

Multivariate analysis in Table 6 revealed a statistical association between accuracy of test performance and the practice of recording all QA elements such as client demographics, kit names, lot numbers, expiration dates, tester name, and individual and final HIV results on the HTS registers for participants who sometimes record (95% CI: 1.45–16.74, *P* = 0.011) and those who don’t record at all (95% CI: 1.24–135.17, *P* = 0.033) compared with those who always record.

**TABLE 6:** Association of practice characteristics and accuracy of rapid HIV test performance.

Characteristic	Univariate analysis			Multivariate analysis		
	cOR	95% CI	*P*	aOR	95% CI	*P*
**Recording all the QA elements in the HIV Testing Services (HTS) register**						
Yes†	-	-	-	-	-	-
No	6.00	0.72–49.84	0.097	12.93	1.24–135.17	0.033*
Sometimes	2.67	1.37–5.18	0.004*	4.93	1.45–16.74	0.011*
**Frequency of participation in Independent Quality Control (IQC) testing**						
As per frequency as stated in QA guidelines†	-	-	-	-	-	-
Sometimes	1.55	0.88–2.72	0.127	0.34	0.10–1.09	0.070
Not at all	1.50	0.42–5.32	0.530	0.44	0.09–2.20	0.316
**Frequency of participation in Proficiency Testing (PT)**						
Once a year	1.13	0.43–2.92	0.808	0.73	0.22–2.38	0.603
As stated in QA guidelines (twice a year)	1.00	-	-	1.00	-	-
**What happens after receiving unacceptable (false) Independent Quality Control (IQC) results**						
Implement and document corrective action†	-	-	-	-	-	-
I don’t implement corrective action	1.42	0.68–2.97	0.356	1.86	0.66–5.28	0.241
I am not sure	3.14	1.34–7.36	0.008*	1.28	0.40–4.15	0.679

aOR, adjusted odds ratio; cOR, crude odds ratio; CI, confidence interval; QA, quality assurance.

* , Significant at the 0.05 level.

† , Reference group; no values are generated when logic regression model is performed.

However, there was no statistical association between accuracy of test performance and the practice of IQC testing as per frequency recommended in QA guidelines (95% CI: 0.10–1.09, *P* = 0.070). Similarly, a statistical association was not observed between accuracy of test performance and participation in PT test as per the recommendations. In addition, a statistical association was not observed between accuracy of test performance and procedure when receiving unacceptable IQC results.

## Discussion

The aim of this cross-sectional study was to determine the factors associated with test performance accuracy of rapid HIV testing among testers in healthcare facilities in a district in the Eastern Cape that have received HIV QA training. The study also attempted to determine the testers’ characteristics for knowledge level, perceived attitude and the practices associated with performance of rapid HIV tests.

The main outcome of the study objective revealed that the prevalence of accuracy in rapid HIV test performance was improved when testers were exposed to rapid HIV QA training. Tester accuracy levels measured by PT score of greater than 80% were achieved among 68.2% of the testers. Comprehensive HIV QA training was significantly associated with the accuracy of test performance (*P* = 0.001).

The level of education was weakly associated with accuracy of HIV test results. Particularly, having a Grade 12 or Matric level of education was negatively associated with accuracy of test performance (*P* = 0.017). It is evident from the results that lay counsellors are important in the provision of HTS. However, the limited resources that are mainly characterised by shortages in the number of adequately qualified personnel can make the rollout of task-shifting complicated. Results from other studies also reveal that, because of lower levels of education, the quality of results offered by lay counsellors may be doubted.^15,16^ Therefore, more training efforts should be focused on these testers to ensure higher degrees of accuracy. Rapid HIV tests are simple to perform; however, they may also be complex and require technical understanding when testing is performed by non-laboratory personnel such as lay counsellors. Challenges have been seen on the quality of testing and accuracy of test results in these instances.^15.^ Furthermore, a study by Mwisongo et al. noted that testers with a higher level of education than Grade 11 seemed to understand QA of testing concepts better than the lower grades of education.^14^

We found an association between accuracy and rural geographic location of participants, with participants in rural facilities showing lower test accuracy compared to urban facilities (*P* = 0.021). Rural health facilities are staffed with limited skilled personnel compared to urban clinics.^17^ Furthermore, the proximity of many rural facilities is remote, and this may pose challenges to the quality of services with regard to accessing facilities for training and mentorship. Subsequently, it is assumed that facility HIV test performance and accuracy of results will be compromised in these settings. This was revealed by the district score of 61.5% against a national of 78.4% in the recent national assessments for QA of rapid HIV testing and, by contrast, the staff training and competency domain score was lower in the Eastern Cape (40%) when compared to the national score (61.2%).^6^

Study data did not reveal a statistical difference when comparing accuracy of test performance between nurse clinicians and counsellors. This is in concordance with other studies, which have shown that counsellors will improve testing techniques extensively when they test routinely as part of the task-shifting role. In a study conducted in rural KZN, rapid HIV test results produced by counsellors were seen to be 98% concordant when compared to those produced in the laboratory by highly skilled laboratory personnel.^9^ Another study that evaluated quality of testing of home-based rapid HIV testing in a rural district in Malawi showed a sensitivity rate of 99.6%, and 100% specificity, following training of lay counsellors.^18^

An important indicator of accuracy was having received the standardised and comprehensive training on QA of rapid HIV testing. Similarly, a previous HTS study conducted in South Africa found that with more comprehensive training and practical sessions, lay counsellors could conduct testing processes and achieve high PT scores.^6^ This is also seen in numerous published literature, where it is stated that various factors and practices that raise doubt about the quality of rapid HIV testing in non-laboratory settings may be a result of a lack of knowledge of HIV testing procedures and guidelines, and that adherence to testing procedures should improve the level of accuracy of test performance because the testers would have been exposed to comprehensive training.^18,19,20,21^

Knowledge reflects the capacity for one to imagine and perceive issues and, therefore, the knowledge of QA processes is advantageous to the outcome of accurate and reliable HIV results. Our analyses showed that there were statistical differences seen in some of the knowledge characteristics in association with accuracy of test performance. Those who responded that they were not sure when they received mentorship or technical assistance (TA) were significantly less likely to achieve test accuracy (*P* = 0.008). The International Centre for AIDS Care and Treatment Programme (ICAP) has defined TA in the public health setting to include coaching, mentoring, and in-service training.^22,23^ Exposure to mentorship aimed at focused in-service training has been proven to assist testers with confidence in task performance.

We also found that if implementation of QA processes was viewed as an extra burden of work, there was a statistical association with accuracy of test performance (*P* = 0.022). This contrasts with previous studies which reported that with the implementation of task-shifting, the high workload reported among some testers, in particular lay counsellors, was reported to impede the quality of testing because, traditionally, testing was performed by laboratory personnel and lay counsellors were responsible for performing only counselling.^14^

Both knowledge and attitude model the outcomes of practice. Therefore, good knowledge is closely related with good attitude, which will ultimately yield good practice of a particular process. Study data have shown that compliance to recording all the QA elements in the HTS register, such as client demographics, kit names, lot numbers, expiration dates, tester name, and individual and final HIV results, was found to be associated with accuracy of test performance. According to the WHO and Jaya et al., the main factors affecting the accuracy of HIV test performance include the quality of the HIV rapid test kits, and user error occurring during the testing and post-testing phase.^7,8^ Good record keeping is important for monitoring of the performance of testing sites, testers, and the validity of testing algorithm through the collection and analysis of routine HIV testing data. Standardised registers for HIV testing sites should be used as ongoing quality monitoring tools, particular in resource-limited settings. Several studies have focused on strengthening of good record keeping as a key component of ensuring quality of rapid HIV testing.^24,25,26^

### Limitations

A particular limitation to the study was the sample size, in that the PT results available at the facility level may not have been a complete representation of all testers available at facility level, because each PT survey may be different from the previous surveys and different testers may have participated as a result of high facility personnel turnover. The study thus focused on the last PT survey results of 2021. An ideal study design would have been a cohort design to allow the researcher to follow the same testers and assess accuracy over time, and therefore measure the outcome of QA implementation in facilities. The study used available records at the facility level to verify PT score results, which is then used for programmatic purposes, and this posed additional limitations because the researcher was restricted to the usage of the available data. An additional limitation is the bias in SAQ because of the lack of guidance to participants during the interview, and some of the branching questions could have been challenging to respond to.

Furthermore, the findings of this study may not be generalisable to other settings because the participants were chosen using a purposive sampling technique, and non-probability sampling methods often result in biased samples.

## Conclusion

The study showed an improvement in prevalence of accuracy of rapid HIV test performance among PHC workers that have been exposed to HIV rapid test QA training and provided with consistent mentorship. In addition to training, the study revealed a negtive association between accuracy of test performance and rural geographical location of the healthcare facility. It is important to recognise that the association between HCW characteristics in terms of knowledge, attitude, and practice/behaviour are complex, and should be considered when developing training policies. In addition, this is more relevant to consider when investigating effectiveness of any training programme. The important driver of both attitude and practice is knowledge; therefore, the degree of knowledge was assessed in certain areas where training of QA of rapid HIV testing remains a challenge. Further research will allow for nationwide expansion and continuous development of a National QA Training Curriculum on behalf of the NDOH to support the national scale-up and roll-out of a QA programme.
